# Supplementary material for: COZOID: contact zone identifier for visual analysis of protein-protein interactions
Source: BMC Bioinformatics. 2018 Apr 6;19:125. doi: 10.1186/s12859-018-2113-6 (PMC5889581; doi:10.1186/s12859-018-2113-6)
Supplement: Supplementary file 2 — Software build. Executable binary file of the software tool. (ZIP 118,784 kb) [file 12859_2018_2113_MOESM2_ESM.zip › cozoid/java/docs/beansbinding-1.2.1-doc.zip!/org/jdesktop/swingbinding/adapters/package-summary.html]

org.jdesktop.swingbinding.adapters (Beans Binding)


---


|  |  |  |
| --- | --- | --- |
| **Overview** \| **Package** \| Class \| **Use** \| **Tree** \| **Deprecated** \| **Index** \| **Help** | |  |
| **PREV PACKAGE**   **NEXT PACKAGE** | **FRAMES**    **NO FRAMES**     **All Classes** |  |


---

## Package org.jdesktop.swingbinding.adapters

Private implementation details for Swing binding.

**See:**
  
          **Description**

| **Class Summary** | |
| --- | --- |
| **AbstractButtonAdapterProvider** |  |
| **AbstractButtonAdapterProvider.Adapter** |  |
| **BeanAdapterBase** |  |
| **JComboBoxAdapterProvider** |  |
| **JComboBoxAdapterProvider.Adapter** |  |
| **JListAdapterProvider** |  |
| **JSliderAdapterProvider** |  |
| **JSliderAdapterProvider.Adapter** |  |
| **JSpinnerAdapterProvider** |  |
| **JSpinnerAdapterProvider.Adapter** |  |
| **JTableAdapterProvider** |  |
| **JTextComponentAdapterProvider** |  |

## Package org.jdesktop.swingbinding.adapters Description

Private implementation details for Swing binding. Contains adapters
for certain Swing classes to adapt existing properties of
interest to the Beans specification, and provide additional
synthetic properties. These classes are public as required to enable the
properties to be inspected at runtime. Nonetheless, they are an
implementation detail of the Swing binding support. Do not access them
directly as they may change or go away at any time.

For the public list of interesting Swing properties, see the
swingbinding package level
documentation.

---


|  |  |  |
| --- | --- | --- |
| **Overview** \| **Package** \| Class \| **Use** \| **Tree** \| **Deprecated** \| **Index** \| **Help** | |  |
| **PREV PACKAGE**   **NEXT PACKAGE** | **FRAMES**    **NO FRAMES**     **All Classes** |  |


---
